# *Palladin* Mutation Causes Familial Pancreatic Cancer: Absence in European Families

**DOI:** 10.1371/journal.pmed.0040164

**Published:** 2007-04-24

**Authors:** Emily Slater, Vera Amrillaeva, Volker Fendrich, Detlef Bartsch, Julie Earl, Louis J Vitone, John P Neoptolemos, William Greenhalf

We read with interest the article published in *PLoS Medicine* by Pogue-Geile et al. [1] reporting an apparent mutation in the KIAA0992 splice variant of the *palladin* gene in a family previously reported to have a high incidence of pancreatic cancer. Pogue-Geile and others had previously established that the 4q32–34 locus segregated with pancreatic cancer in this family by screening for pre-neoplastic lesions, which could then be used as a marker for mutation carriers [2]. In the *PLoS Medicine* paper the authors show that the mutation in *palladin* is on the 4q32–34 haplotype that segregates with the disease. The European Registry of Hereditary Pancreatitis and Familial Pancreatic Cancer (EUROPAC) and the German National Case Collection for Familial Pancreatic Cancer (FaPaCa) have recently shown that a mutation on 4q32–34 is unlikely to explain pancreatic cancer in a majority of our European families, but we did not rule out segregation with the disease in a minority of families [3].

Naturally we were keen to establish if the mutation seen in Family X from America was seen in any of our families, and so we have sequenced the locus in 74 individuals who were either affected by pancreatic cancer or who are obligate carriers (assuming autosomal dominant inheritance) of the disease mutation (in 74 families). We have also sequenced the locus in 14 affected individuals from 14 families with familial multiple mole melanoma with cases of pancreatic cancer (FAMMM-PC) [4] and nine sporadic pancreatic cancer patients of less than 50 years of age. We did not identify the mutation in any of the individuals, neither as a heterozygote or a homozygote.

This does not of course mean that other mutations in coding or non-coding regions of this variant of *palladin* or other variants are absent from European families. However, it is noteworthy that the phenotype of Family X is significantly different from the phenotype common to the families on the EUROPAC/FaPaCa registries. In particular, the incidence of diabetes in our families is relatively low, except where the diabetes is a direct consequence of development of cancer [3]. This presentation contrasts strongly with the family harbouring the *palladin* mutation [1,2], where diabetes was common. It is possible that Family X (and the association with *palladin* mutation) is not typical of the familial pancreatic cancer syndrome.
